# Monitoring Adherence to Asthma Inhalers Using the InspirerMundi App: Analysis of Real-World, Medium-Term Feasibility Studies

**DOI:** 10.3389/fmedt.2021.649506

**Published:** 2021-07-15

**Authors:** Cristina Jácome, Rute Almeida, Ana Margarida Pereira, Rita Amaral, Pedro Vieira-Marques, Sandra Mendes, Magna Alves-Correia, José Alberto Ferreira, Inês Lopes, Joana Gomes, Luís Araújo, Mariana Couto, Cláudia Chaves Loureiro, Lilia Maia Santos, Ana Arrobas, Margarida Valério, Ana Todo Bom, João Azevedo, Maria Fernanda Teixeira, Manuel Ferreira-Magalhães, Paula Leiria Pinto, Nicole Pinto, Ana Castro Neves, Ana Morête, Filipa Todo Bom, Alberto Costa, Diana Silva, Maria João Vasconcelos, Helena Falcão, Maria Luís Marques, Ana Mendes, João Cardoso, José Carlos Cidrais Rodrigues, Georgeta Oliveira, Joana Carvalho, Carlos Lozoya, Natacha Santos, Fernando Menezes, Ricardo Gomes, Rita Câmara, Rodrigo Rodrigues Alves, Ana Sofia Moreira, Carmo Abreu, Rui Silva, Diana Bordalo, Carlos Alves, Cristina Lopes, Luís Taborda-Barata, Ricardo M. Fernandes, Rosário Ferreira, Carla Chaves-Loureiro, Maria José Cálix, Adelaide Alves, João Almeida Fonseca

**Affiliations:** ^1^Center for Health Technology and Services Research (CINTESIS), Faculty of Medicine, University of Porto, Porto, Portugal; ^2^Department of Community Medicine, Information and Health Decision Sciences (MEDCIDS), Faculty of Medicine, University of Porto, Porto, Portugal; ^3^Allergy Unit, Instituto and Hospital CUF, Porto, Portugal; ^4^Department of Cardiovascular and Respiratory Sciences, Porto Health School, Polytechnic Institute of Porto, Porto, Portugal; ^5^Department of Women's and Children's Health, Pediatric Research, Uppsala University, Uppsala, Sweden; ^6^Serviço de Imunoalergologia, Unidade I, Centro Hospitalar Vila Nova de Gaia/Espinho, Vila Nova de Gaia, Portugal; ^7^Serviço Pneumologia, Hospitais da Universidade de Coimbra, Coimbra, Portugal; ^8^Serviço de Pneumologia, Hospital Distrital da Figueira da Foz, Figueira da Foz, Portugal; ^9^Serviço de Imunoalergologia, Centro Hospitalar e Universitário de Coimbra, Coimbra, Portugal; ^10^Imunoalergologia, Centro Hospitalar de Leiria, Leiria, Portugal; ^11^Serviço de Pediatria, Centro Materno Infantil do Norte, Centro Hospitalar Universitário do Porto, Porto, Portugal; ^12^Serviço de Imunoalergologia, Hospital de Dona Estefânia, Centro Hospitalar Universitário de Lisboa Central, Lisboa, Portugal; ^13^Serviço de Imunoalergologia, Hospital Infante D. Pedro, Centro Hospitalar Baixo Vouga, Aveiro, Portugal; ^14^Serviço de Pneumologia, Hospital Beatriz Ângelo, Loures, Portugal; ^15^Serviço de Pediatria, Hospital da Senhora da Oliveira, Guimarães, Portugal; ^16^Serviço de Imunoalergologia, Centro Hospitalar Universitário de São João, Porto, Portugal; ^17^Serviço de Imunoalergologia, Centro Hospitalar Universitário do Porto, Porto, Portugal; ^18^Serviço de Imunoalergologia, Hospital de Santa Maria, Centro Hospitalar Universitário Lisboa Norte, Lisboa, Portugal; ^19^Serviço de Pneumologia, Hospital Santa Marta, Centro Hospitalar Universitário de Lisboa Central, Lisboa, Portugal; ^20^Serviço de Pediatria, Hospital Pedro Hispano, Unidade Local de Saúde de Matosinhos, Matosinhos, Portugal; ^21^Serviço de Imunoalergologia, Hospital Amato Lusitano, Unidade Local de Saúde de Castelo Branco, Castelo Branco, Portugal; ^22^Serviço de Imunoalergologia, Centro Hospitalar Universitário do Algarve, Portimão, Portugal; ^23^Serviço de Pneumologia, Hospital Garcia de Orta, Almada, Portugal; ^24^Serviço de Imunoalergologia, Serviço de Saúde da Região Autónoma da Madeira, Funchal, Portugal; ^25^Serviço de Imunoalergologia, Hospital do Divino Espírito Santo, Ponta Delgada, Portugal; ^26^Serviço de Imunoalergologia, Hospital São Pedro de Vila Real, Centro Hospitalar De Trás-Os-Montes E Alto Douro, Vila Real, Portugal; ^27^Serviço de Pediatria, Unidade Hospitalar de Famalicão, Centro Hospitalar do Médio Ave, Vila Nova de Famalicão, Portugal; ^28^Serviço de Pneumologia, Hospital Nossa Senhora do Rosário, Centro Hospitalar Barreiro Montijo, Barreiro, Portugal; ^29^Unidade de Imunoalergologia, Hospital Pedro Hispano, Unidade Local de Saúde de Matosinhos, Matosinhos, Portugal; ^30^Imunologia Básica e Clínica, Faculdade de Medicina, Universidade do Porto, Porto, Portugal; ^31^Department of Allergy and Clinical Immunology, Cova da Beira University Hospital Center, Covilhã, Portugal; ^32^CICS - Health Sciences Research Center, University of Beira Interior; NuESA –Environment and Health Study Group, Faculty of Health Sciences, Covilhã, Portugal; ^33^Departamento de Pediatria, Hospital de Santa Maria, Centro Hospitalar de Lisboa Norte, Lisboa, Portugal; ^34^Serviço Pediatria Ambulatória, Centro Hospitalar e Universitário de Coimbra, Coimbra, Portugal; ^35^Serviço de Pediatria, Hospital de São Teotónio, Centro Hospitalar Tondela–Viseu, Viseu, Portugal; ^36^Serviço de Pneumologia, Unidade I, Centro Hospitalar Vila Nova de Gaia/Espinho, Vila Nova de Gaia, Portugal; ^37^MEDIDA – Medicina, Educação, Investigação, Desenvolvimento e Avaliação, Porto, Portugal

**Keywords:** mHealth, smartphone, technology assessment, medication adherence, self-management, patient participation

## Abstract

**Background:** Poor medication adherence is a major challenge in asthma and objective assessment of inhaler adherence is needed. InspirerMundi app aims to monitor inhaler adherence while turning it into a positive experience through gamification and social support.

**Objective:** We assessed the medium-term feasibility of the InspirerMundi app to monitor inhaler adherence in real-world patients with persistent asthma (treated with daily inhaled medication). In addition, we attempted to identify the characteristics of the patients related to higher app use.

**Methods:** Two real-world multicenter observational studies, with one initial face-to-face visit and a 4-month telephone interview, were conducted in 29 secondary care centers from Portugal. During an initial face-to-face visit, patients were invited to use the app daily to register their asthma medication intakes. A scheduled intake was considered taken when patients took a photo of the medication (inhaler, blister, or others) using the image-based medication detection tool. Medication adherence was calculated as the number of doses taken as a percentage of the number scheduled. Interacting with the app ≥30 days was used as the cut-off for higher app use.

**Results:** A total of 114 patients {median 20 [percentile 25 to percentile 75 (P25–P75) 16–36] years, 62% adults} were invited, 107 (94%) installed the app and 83 (73%) completed the 4-month interview. Patients interacted with the app for a median of 18 [3–45] days, translated on a median use rate of 15 [3–38]%. Median inhaler adherence assessed through the app was 34 [4–73]% when considering all scheduled inhalations for the study period. Inhaler adherence assessed was not significantly correlated with self-reported estimates. Median adherence for oral and other medication was 41 [6–83]% and 43 [3–73]%, respectively. Patients with higher app use were slightly older (*p* = 0.012), more frequently taking medication for other health conditions (*p* = 0.040), and more frequently prescribed long-acting muscarinic antagonists (LAMA, *p* = 0.024). After 4 months, Control of Allergic Rhinitis and Asthma Test (CARAT) scores improved (*p* < 0.001), but no differences between patients interacting with the app for 30 days or less were seen.

**Conclusions:** The InspirerMundi app was feasible to monitor inhaler adherence in patients with persistent asthma. The persistent use of this mHealth technology varies widely. A better understanding of characteristics related to higher app use is still needed before effectiveness studies are undertaken.

## Introduction

Adherence to long-term therapy for chronic illnesses is estimated to reach only 50% on average (1). Current research shows that non-adherence is associated with almost 200,000 deaths and €80–125 billion costs in the European Union (2), and with 125,000 deaths per year and up to $300 billion costs every year in the United States (3). Non-adherence in medication constitutes, thus, a global and complex health problem contributing to increased economic burden and poor health outcomes.

Asthma is among the chronic conditions with the highest rates of nonadherence, together with cancer, diabetes, epilepsy, HIV/AIDS, and hypertension (1, 4). As with other diseases, daily medications are critical for asthma effective treatment and managing comorbidities (5). Yet, poor medication adherence is a major challenge in asthma (6, 7), associated with the complexity of the inhaled regimen (inhaler handling, inhalation technique).

Interventions to tackle medication non-adherence in the last decades have led to little improvement, mostly being complex to implement in real-life practice (8). mHealth technologies, with their widespread use, are promising to empower patients in detecting and managing non-adherence in real-world clinical practice (9). Around 1,500 mobile apps are targeting patients with asthma in app stores (10). Yet, these asthma apps capture < 1% of the target market, because most apps are exclusively tracker apps, do not provide behavior change support, and do not enable automated data input or personalized feedback (10). Another explanatory reason is that apps are generally developed and poorly grounded in clinical research and practice.

An InspirerMundi app is an innovative tool developed to measure and improve medication adherence in patients with asthma. The app aims to transform adherence to inhalers into a positive experience through gamification and social support while allowing for objective monitoring of inhaler adherence. Gamification has been used to promote the use of asthma apps (11). The concept and gameplay of the InspirerMundi app are derived from the social interaction of promoting users, in a quest to help other users to increase inhaler adherence (while being an example of good adherence, an “Inspirer”). “Inspirers” can increase the sphere of positive influence by “coaching” an expanding network of users- “Warriors” (that, as they progress and keep good medication adherence, can become “Inspirers” themselves). The most innovative app technology is the medication detection tool based on advanced processing of inhaler images captured with the smartphone camera (12), which aims to offer an objective adherence measure that can be easily integrated into the lives of patients. It is believed that the app is suitable to be used by real-world patients as its development was grounded in previous research and cooperation with patients with asthma and physicians (13). Yet, this needs to be demonstrated in real-world feasibility studies.

Thus, we aimed to evaluate the medium-term feasibility of the InspirerMundi app among real-world patients with asthma to monitor inhaler adherence. In addition, we attempted to identify the characteristics of patients related to higher app use.

## Methods

### Study Design

This study includes data from two real-world multicenter observational studies with a similar design: one initial face-to-face medical visit and a 4-month telephone interview (14). During the studies, there were two additional telephone interviews: at 1 week and 1 month. Patients with persistent asthma were recruited, in one study between November 2017–June 2018 and in the other between October 2019–January 2020, at 29 allergy, pulmonology, and pediatric secondary care centers in Portugal (North, Center, Lisbon, Algarve, Azores, and Madeira regions). The protocol was approved by the ethics committees of all the participating centers. The studies were conducted according to the ethical standards established in the Declaration of Helsinki. Eligible patients were approached by physicians during medical visits and invited to participate in a study on the use of an app to register their asthma medication daily intakes. Written informed consent was obtained before enrolment in the study. Adult patients signed a consent form; adolescents signed an assent form, and a parental consent form was also obtained. This study is reported according to STROBE (Strengthening the Reporting of Observational Studies in Epidemiology) guidelines (15).

### Participants

Patients were included if they (1) had a previous medical diagnosis of persistent asthma (defined by the need for a daily inhaled controller medication), (2) were at least 13 years old, and (3) were able to use mobile applications and had access to a mobile device with the Internet. All inhaled controller treatments were allowed, and there was no change in any prescribed medication as a result of the participation in this study. Patients were excluded if they had a diagnosis of a chronic lung disease other than asthma or a diagnosis of another significant chronic condition with possible interference with the aim of the study.

### InspirerMundi App

**Figure 1:**
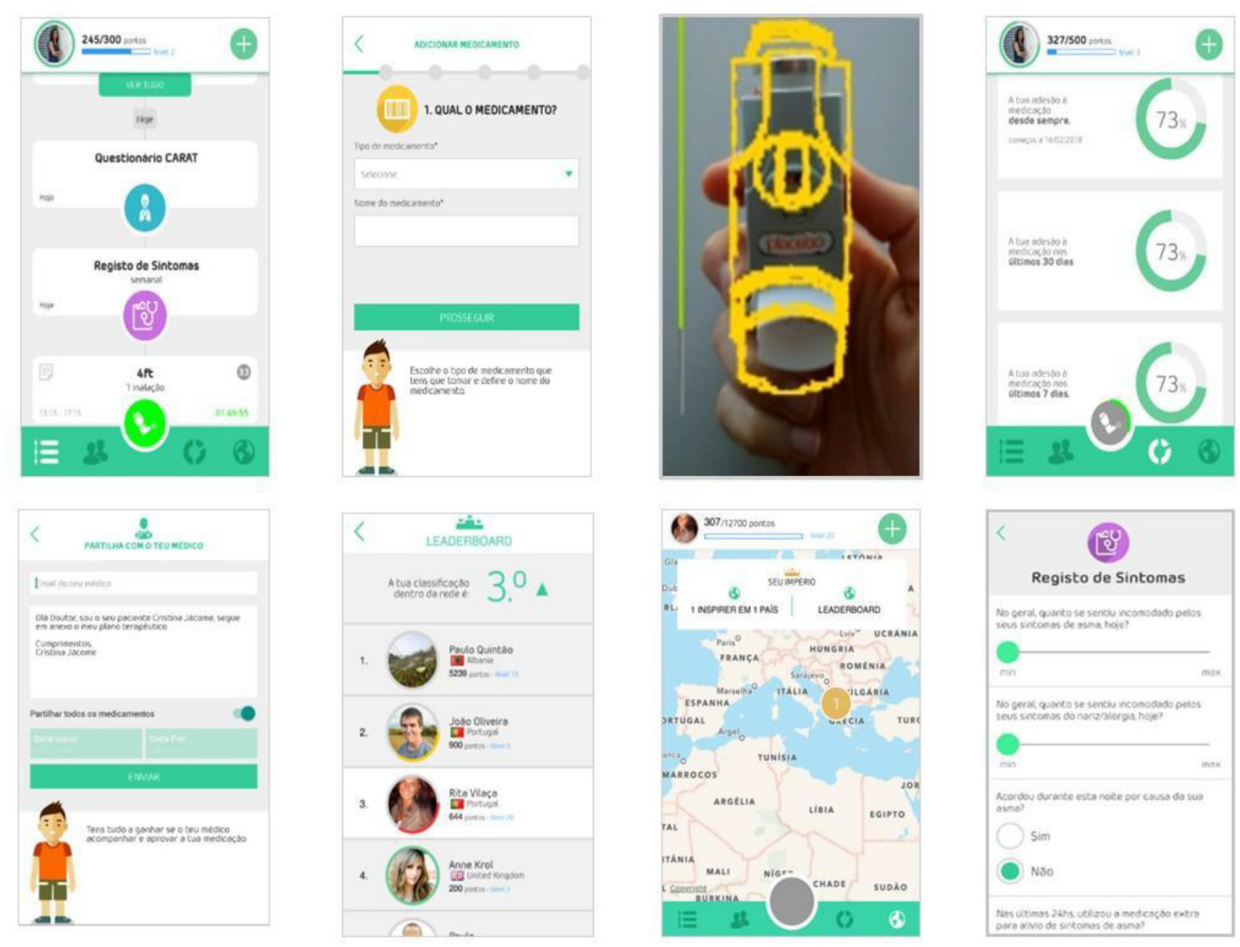
InspirerMundi app screenshots (version 1.1).

InspirerMundi app is focused on supporting medication management of the patients and on promoting treatment adherence. The app includes the registration of the therapeutic plan, focused on asthma medication, but patients were free to insert medications for other conditions (e.g., nasal sprays for allergic rhinitis, etc.). After registering the plan and scheduling medications, the app activates notifications when a medication is due and allows the confirmation of performed intakes through a medication detection tool that uses the smartphone camera. The medication detection tool is based on advanced image processing techniques and confirms inhalers with dose counter through template matching (when taking the photo, the user needs to align his/her inhaler with the template shown on the screen, chosen based on the inhaler registered) (13). In this feasibility study, only the inhaler type was confirmed, but in the future, this tool will aim to automatically read the number in the dose counter (objective verification of adherence). The medication detection tool is also applied to other types of inhalers or other drug formulations (e.g., blister or other recipients), but not the template matching feature. In the tested app version, a scheduled intake/dose was considered to have been taken when patients took a photo of the medication (inhaler, blister, or other recipients, as can be seen in the third screenshot in Figure 1) using the image-based medication detection tool. Besides planned medication, the user may also insert events of relief medication intake that are not considered for treatment adherence assessment. Physician involvement is also considered by providing the user with the possibility of sharing the registered therapeutic plan and respective adherence data. Medication management is supported by a timeline that reflects the registered therapeutic plan. The timeline also includes events related to gamification and symptom monitoring. Patients are invited to answer three types of questionnaires related to their symptoms and asthma control: daily questionnaire (daily after 6 p.m., six questions related to symptom burden), weekly questionnaire (once a week, four questions related to symptom burden and daily living impact), and CARAT (16) (by default once a month, but can be personalized to weekly or every 2 weeks). The concept and gamification dynamics are twofold: achievement challenges and social interaction. Gameplay and mechanics included in the app derive from achievements, such as points/badges, promotion of social interaction, and based on a story of evolving from a “Warrior” (beginner player, level 1) to an “Inspirer” (advanced player, an example of good adherence, available from level 10 onwards) that can motivate “Warriors” to improve adherence. The main components that support the game are points earned when users take certain actions, such as registering a new medication, registering scheduled medication intakes at the right time, answering symptom questionnaires, or getting a positive assessment from other users in their network. Registering events of relief medication intake does not generate additional points. Another encouragement for the interaction of users is the attribution of virtual badges (12 available). Whenever the user reaches a certain goal (e.g., “Role Model” badge when the user links to the first “Warrior”) or does something special (e.g., “Big Influencer” badge when the user reaches five “Warriors”), he/she will be rewarded and get a virtual badge for such actions. The app, developed in Portuguese, English, and Spanish, was released on Apple App Store (version 1.1) and Google Play store (versions 1.1.x and 1.2), being available for download in Portugal, and Spain only.

### Data Collection

Feasibility outcomes included study retention, app installation, and use (i.e., days of use).

During the initial face-to-face visit, physicians reported asthma control of patients according to the Global Initiative for Asthma (GINA) (5); last known value of percent predicted Forced Expiratory Volume in the first second (FEV_1_); the number of exacerbations in the previous year [defined as episodes of progressive increase in shortness of breath, cough, wheezing, and/or chest tightness, requiring a change in maintenance therapy (17)]; and the number of unscheduled medical visits (primary care, secondary care, or emergency department). Physicians also reported the current asthma treatment of patients, including inhaled and oral medication, allergen immunotherapy, and biologic therapy. At the visit, patients answered written questionnaires on demographic data (age, sex, body mass index, and smoking habits), adherence to inhaled controller asthma medication during the previous week [using a 100 mm visual analog scale (VAS) (18)], asthma control during the previous 4 weeks [using CARAT (16)], perception of their overall health [using the EuroQol five-dimensional (EQ-5D) VAS (19)], and previous use of Health and Fitness and asthma apps.

Approximately 1 week later, through a telephone interview, the Morisky Medication Adherence Scale (MMAS-4) was applied (20). It contains four questions with yes (0) or no (1) answers. The item scores are summed to define three levels of adherence: 0 (high adherence), 1–2 (medium adherence), and 3–4 (low adherence). Patients were also asked if they were also taking medication for other health conditions. At a 4-month follow-up, patients were asked in a telephone interview about their adherence to inhaled controller medication during the previous week (on a 0–100 scale), and their asthma control with CARAT. Interviews were performed by a central team of trained health professionals.

### Data Analysis

Descriptive statistics were used to characterize the sample and app use. The normality of each variable was investigated with Kolmogorov-Smirnov tests and visual analysis of histograms. The app use rate was calculated by dividing the number of days with app use by the total study period (120-day period). Adherence to medication measured by the app was calculated as the number of doses taken (as registered on the image-based medication tool) as a percentage of the total number of doses scheduled (that should have been expected to have been taken based on the registered action plan). Two methods were used for this calculation, the first method considered the medication (taken and scheduled) only on days with app use, and the second method considered the medication taken regardless of app use, i.e., considering all days with medication scheduled during the study period. We performed a preliminary, exploratory analysis to identify the characteristics of patients related to a higher app use (i.e., interacting with the app for at least 30 days) vs. patients with lower interaction/no interaction using Mann-Whitney U tests for continuous variables and chi-square tests for categorical variables. Spearman correlations and scatterplots were used to explore relationships between self-reported adherence (at baseline and 4 month) and adherence measured with the app during the study period. Two-way ANOVA with repeated measures was used to assess the effects of time (4 month), group (patients interacting with the app for at least 30 day vs. patients with lower interaction/no interaction), and these factors in combination on CARAT total score. The proportion of patients with the controlled disease was compared between baseline and 4 month using the McNemar's test. Statistical analyses were performed using IBM SPSS Statistics V.26.0 (IBM Corporation, Armonk, NY, USA). The level of significance was set at 0.05.

## Results

A total of 114 patients (62% adults) were enrolled, most recruited from allergy centers (63%), followed by pulmonology (21%) and pediatric (16%) centers. Most patients were on inhaled corticosteroids/long-acting beta-agonists (ICS/LABA) combination therapy (83%), used only one inhaler (62, 94% of those on maintenance and reliever therapy-MART regimen), and reported high inhaler adherence (median VAS 84 [percentile 25 to percentile 75, P25–P75 65–95] mm). Based on the GINA classification, 55% of the participants had their asthma well-controlled. Based on CARAT, the proportion of controlled disease was 23%. Characteristics of the participants are shown in Table 1.

**Table 1:** The baseline characteristics of participants (*n* = 114).

	**Total (*n* = 114)**	**App used <30 days (*n* = 72)**	**App used ≥30 days (*n* = 42)**	***p*-value**
Age, median (P25–P75), years	20 (16–36)	19 (15–30)	27 (17–42)	0.012
Adults	71 (62%)	42 (58%)	29 (69%)	0.255
Female	71 (62%)	43 (60%)	28 (67%)	0.461
BMI, median (P25–P75), kg/m^2^	23 (21–26)	23 (21–26)	23 (21–27)	0.463
Smoking status				
Never smoker	92 (81%)	58 (81%)	34 (81%)	0.607
Ex-smoker	16 (14%)	9 (13%)	7 (17%)	–
Current smoker	6 (5%)	5 (7%)	1 (2%)	–
FEV1 % predicted, mean (SD)^a^	95 (22)	95 (86–109)	89 (78–104)	0.091
Inhaled medication				
ICS/LABA	95 (83%)	61 (85%)	34 (81%)	0.602
SABA	29 (25%)	19 (26%)	10 (24%)	0.815
ICS	17 (15%)	10 (14%)	7 (17%)	0.688
LAMA	12 (11%)	4 (6%)	8 (19%)	0.024
Single inhaler	71 (62%)	47 (65%)	24 (57%)	0.418
Self-reported inhaler adherence VAS, median (P25–P75), mm	84 (65–95)	80 (60–90)	86 (79–98)	0.094
MMAS-4^b^				
High adherence	5 (4%)	3 (4%)	2 (5%)	0.845
Medium adherence	66 (58%)	38 (53%)	28 (67%)	–
Low adherence	33 (29%)	21 (29%)	12 (29%)	–
Oral medication–anti-leukotrienes	56 (49%)	31 (43%)	25 (60%)	0.090
Allergen immunotherapy	17 (15%)	14 (19%)	3 (7%)	0.078
Biologic therapy	10 (9%)	4 (6%)	6 (14%)	0.124
Medication for other health conditions	35 (34%)	16 (26%)	19 (45%)	0.040
GINA assessment of symptom control^c^				
Well-controlled	63 (55%)	42 (58%)	21 (50%)	0.344
Partly controlled/uncontrolled	50 (44%)	29 (40%)	21 (50%)	–
CARAT				
Total score, median (P25–P75)	21 (16–24)	20 (16–24)	22 (17–24)	0.962
Controlled disease (>24)	26 (23%)	17 (24%)	9 (21%)	0.789
≥1 exacerbation past year	53 (47%)	36 (50%)	17 (41%)	0.475
≥1 unscheduled medical visit past year	28 (25%)	19 (26%)	9 (21%)	0.177
EQ-5D VAS, median (P25–P75)	85 (75–90)	85 (75–90)	84 (70–93)	0.652
Previous use of health and fitness apps	55 (48%)	34 (47%)	21 (50%)	0.734
Previous use of asthma apps	1 (1%)	0	1 (2%)	0.186

*Values are shown as n (%) unless otherwise indicated. BMI, body mass index; CARAT, Control of Allergic Rhinitis and Asthma Test; EQ-5D, EuroQol five-dimensional; ICS, inhaled corticosteroids; LABA, long-acting beta-agonists; LAMA, long-acting muscarinic antagonists; P25–P75, percentile 25 to percentile 75; SABA, short-acting beta-agonists; SAMA, short-acting muscarinic-antagonists; VAS, visual analog scale*.

a *18 missing values*;

b *10 missing values*;

c *1 missing value*.

**Figure 2:**
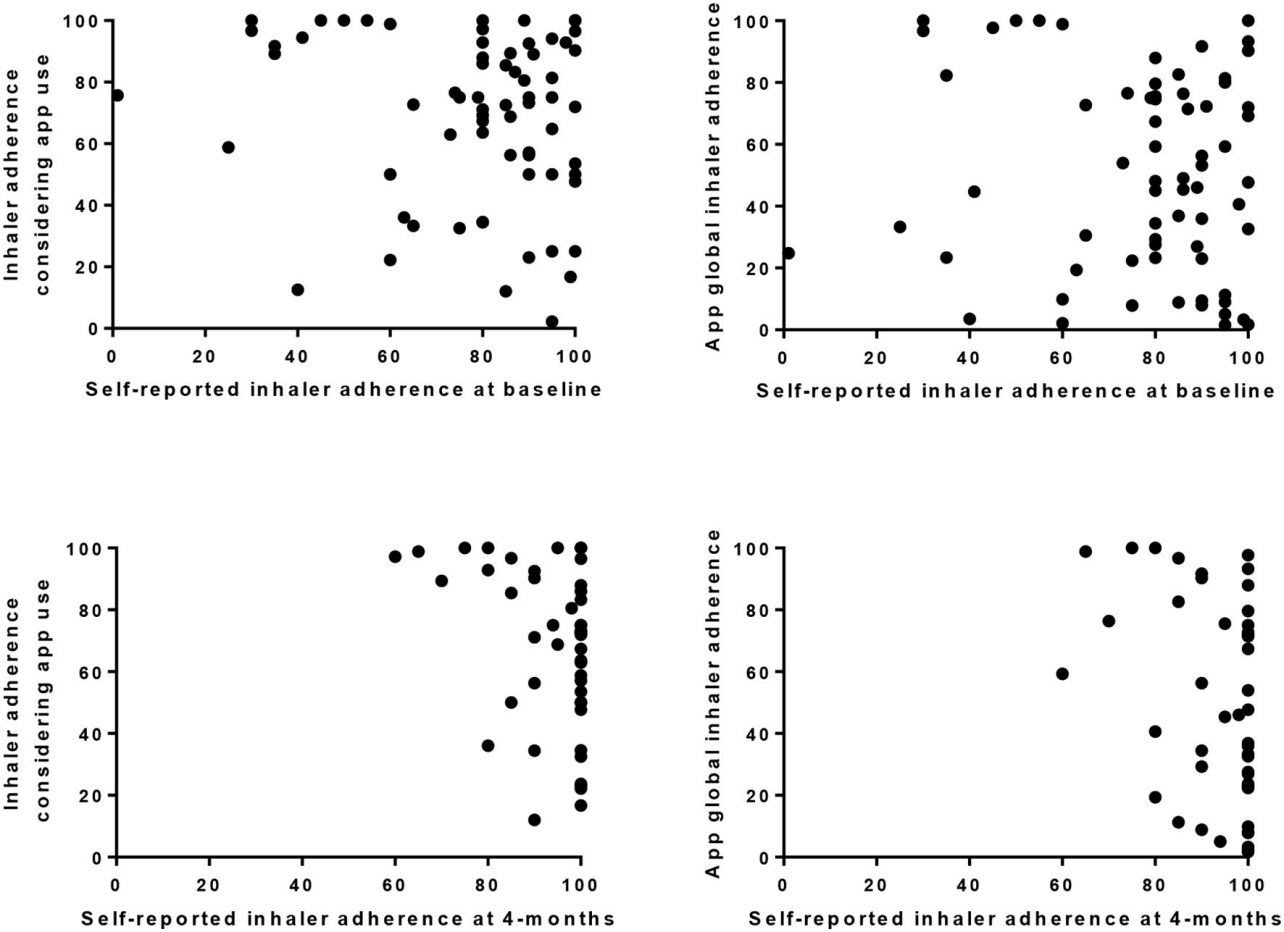
Scatter plots showing the relationship between patients self-reported estimates of inhaler adherence (at baseline *n* = 69 and 4 month *n* = 47), and inhaler adherence calculated using app data with two methods: one method considered the medication taken only on days with app use (Inhaler adherence considering app use), and the other method considered the medication taken regardless of app use, i.e., considering all medication scheduled for the 120 days (app global inhaler adherence).

A total of 107 (94%) patients installed the app, most in the first or second days of the follow-up period [median 1 [0, 8] (range 0–112) days] (Figure 1). About 91 (85%) patients scheduled medication in the app (median 2 [1–3] medications). In 86 (80%), it included at least one inhaler scheduled for 59 [30–100] days. Median inhaler adherence assessed through the app was 67 [23–89]% when considering only days with app use and 34 [4–73]% when considering all scheduled inhalations for the study period. Inhaler adherence assessed with both methods was not significantly correlated with self-reported estimates neither at baseline (84 [65–95], *r* = −1.27 and *r* = −0.109) nor at 4 month (100 [90–100], *r* = −0.253 and *r* = −0.173) (Figure 2).

Forty-eight (45%) patients scheduled at least one oral medication for 30 [17–59] days, and 32 (30%) at least another type of medication (e.g., nasal spray) for 29 [11–40] days. Median adherence to oral and other medication was 57 [33–86] and 44 [6–80]%, respectively, when considering only days with app use; and 41 [6–83] and 43 [3–73]% when considering all scheduled intakes for the study period. Twenty-four patients (22%) registered the use of relief medication for at least 1 day (median 1 [1–2]): 21 (20%) used inhalers and six (6%) other drug formulations (pills, nasal spray).

**Figure 3:**
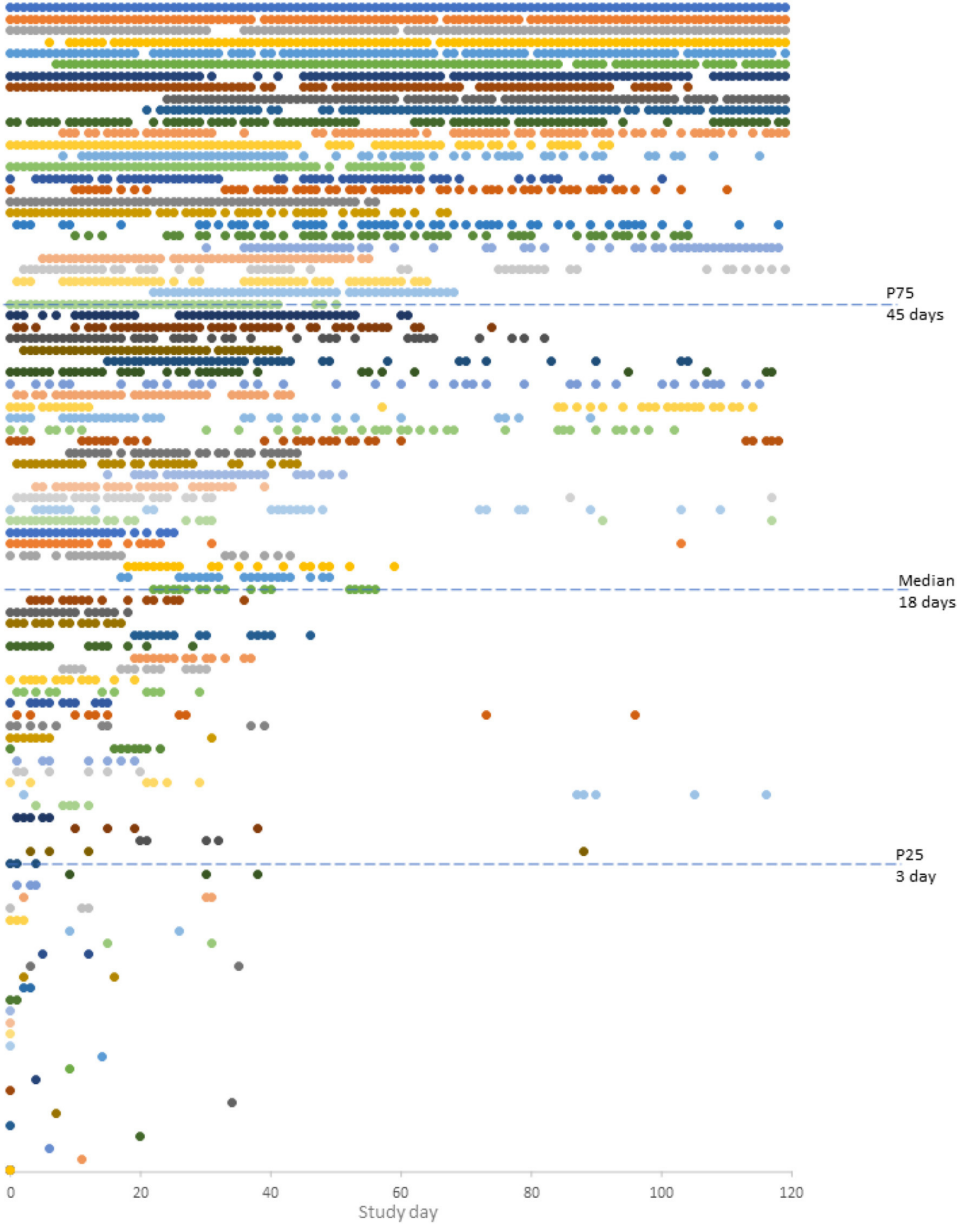
Daily participant engagement with the InspirerMundi app during the 4 month (*n* = 102, five users with missing information). Each circle represents a day with interaction(s) and each color represents each participant.

Patients installing the InspirerMundi app, used it for a median of 18 [3–45] days: 13 (12%) only 1 day, and 42 (39%) for 30 days or more (Figure 3). An overall median use rate of 15 [3–38]% (min–max 1–100%) was observed: being 4 [2–12]% for patients interacting with the app <30 days and 41 [31–65]% for patients interacting 30 days or more. Patients using the app 30 days or more were slightly older, were more frequently taking medication for other health conditions, and were prescribed LAMA. Although not statistically significant, patients using the app 30 days or more seemed to be those with worse FEV_1_ % predicted, that received allergen immunotherapy (current or past), and had higher self-reported inhaler adherence at baseline. As can be seen in Table 1, no other differences were observed.

Eighty-three (73%) patients completed the 4-month interview. After 4 month, CARAT scores improved (*p* < 0.001), with the proportion of patients with controlled asthma increasing from 24 to 57% (*p* < 0.001) (Supplementary Figure 1).

## Discussion

We found that InspirerMundi is feasible to monitor inhaler adherence in real-world patients with persistent asthma that used the app. However, the persistent use of this mHealth technology varies widely. A better understanding of characteristics related to higher app use is still needed before effectiveness studies are undertaken.

Considering that only patients treated with daily inhaled medication were recruited and that they received notifications (at least the 81% that scheduled at least one medication), a higher app use rate was expected. Also, because this app has been developed in close cooperation with patients, based on a user-centered approach (11, 19). This low engagement may be linked to the real-world nature of these studies and the low previous use of asthma apps (1%). Yet, the use rate found is similar to a previous study on an asthma adherence app (median of 17 [6–31] uses in 5 month) (21) and is within the range described in digital health studies (median of 6 days, from 2 to 26, in the first 3 month) (22). The app versions used in these studies were not performing the objective confirmation of the inhaler dose counter. New versions of the app by validating inhaler use through dose tracking may be more attractive to the end-users. Importantly, it may also be related to the low level of maturity of some of the technologies of apps. Moreover, for the same use rate, we found different patterns of app use (e.g., a total of 10 days in a pattern of sequential days vs. a total of 10 days, with intermittent use distributed in 40 days), that should be further explored in future studies with a larger sample. We need to consider that the use rate is negatively influenced by patients using the app for only a few days and we found a much higher use rate (41 [31–65]%) when analyzing patients more persistently using the app. The overall low use rate may also be linked to the real-world nature of the study not restricted to specific non-adherence patterns nor health status. Patients were not selected based on their adherence levels, their self-reported adherence was high, and using MMAS-4 less than one-third were identified as being poorly adherent. Moreover, according to GINA, half had controlled asthma. Future effectiveness studies could thus consider baseline non-adherence and/or uncontrolled asthma as part of the inclusion criteria.

As expected, self-reported inhaler adherence both at baseline and at the 4-month follow-up period overestimated adherence measured with the app. This was somewhat expected as self-reports had a near to perfect treatment adherence behavior, which is unlikely to translate real adherence (18). Median inhaler adherence assessed through the app was 67 [23–89]% when considering only days with app use and 34 [4–73]% when considering all scheduled inhalations for the study period, which is within the adherence range found in clinical trials using electronic trackers (46–88%) (23), and in real-world observational studies (24, 25). But even adherence measured with the app may be overestimating real adherence as validation of inhaler use through dose tracking was not implemented to confirm intake. The accuracy of the adherence estimates is thus expected to improve in future app versions. Twenty-two percent of patients registered the use of relief medication, which seems low when compared with previous asthma research (39–63)% (23). Yet, we cannot assume that all patients that used the app registered the use of relief medication, and this proportion is possibly underestimating real use. But even though, 22% might be a quite high proportion considering that these patients had a recent medical appointment, received telephone calls to monitor their health status, and were using an app to help them to better adhere to the controller inhaler.

Patients interacting with the app for a longer period were slightly older, had LAMA prescribed, and were taking medication for other health conditions. They also tended to be the ones with worse FEV_1_ % predicted, that received allergen immunotherapy, and that had higher self-reported inhaler adherence. Previous studies in asthma and other health conditions show that young and middle-aged adults are more likely to engage with health apps in comparison with older adults (26–28). But comparisons with adolescents were not found. The two age groups included in this study may interact differently with the distinct features of the app, and this may be translated into different app use patterns. For the remaining characteristics, to our knowledge, no previous studies are describing them as relevant factors for asthma app use. Possibly for patients with more complex asthma therapeutic plans and those taking medication for other conditions, the app was perceived as more useful, as they could use the app to better manage treatment adherence. This observation links with a qualitative study that showed that having a consistent routine and planning was considered more relevant by patients with more complex regimens rather than by those with simpler regimens (29). Regarding adherence, a previous study also showed that patients with the highest adherence at baseline were the ones using more frequently an app to improve adherence (21). Possibly, the same personality factors explain adherence behaviors, such as adherence to inhaler adherence or to a mHealth intervention (app). This means that mHealth technologies to improve adherence are not reaching the most relevant target users. The InspirerMundi app is currently being tested in 139 real-world patients recruited at primary care centers, and it will be relevant to verify if these characteristics (age, medication, lung function, and adherence) remain important to explain app interaction, or if others are identified (e.g., sex, asthma control) as observed in other digital studies (27). In the future, the joint analysis of these preliminary studies will enlighten us on the characteristics of patients using this mHealth solution. These characteristics should then be considered in further app developments and in designing mHealth-based interventions to improve adherence to medications.

After the 4-month follow-up period, patients improved their symptoms, as an increase in CARAT scores was observed. This was seen regardless of app use, which may be linked to the possible effect of the medical visit (and related interventions) and the follow-up phone calls at 1 week and 1 month. Nevertheless, we need to consider that data related to the 4-month period is not considering all samples, but only the 73% completing the study (30).

This study has limitations. As this is a preliminary study conducted with a relatively small sample recruited at secondary care centers from Portugal, results may not be generalized to other settings or countries. The number of eligible patients that refused to participate was not collected due to the real-world nature of the study, involving a large number of centers, diverse in terms of setting (public vs. private), dimension, geographical regions, and medical specialties. Considering a similar cohort, in which two-thirds of patients would be interested in using an asthma app and in participating in studies for validation of asthma apps (31), the participation was probably high. Nevertheless, we need to be cautious in this comparison, as the manifestation of interest may be distinct from accepting to participate. Future studies should collect this feasibility outcome. In Portugal, patients with persistent asthma are commonly followed up at secondary care, but can also be followed up by their general practitioner. The InspirerMundi app is thus currently being tested in primary care centers, which will allow us to assess the feasibility in a distinct healthcare setting. Using an app to improve treatment adherence will not be a solution for all patients, as happens with most, if not all, approaches. With this real-life study, together with previous ones and others planned, it should be possible to identify those patients who may benefit most from the InspirerMundi app and to analyze differences in in-app interaction between adolescents and adults. The use of two methods to calculate medication adherence may also be seen as a limitation of the study, yet it was a mitigation plan to overcome the absence of a standardized method to measure adherence (32). The high dropout rate was expected given the real-world nature of the conducted feasibility studies, involving patients from a large number of secondary care centers (29 in total), very diverse in terms of settings (public vs. private), dimension, geographical regions, and of medical specialties (allergy, pulmonology, and pediatrics). But we need to consider that our real-world study contrasts with clinical trials, which are most often conducted in well-selected academic centers, with more homogeneous samples, and with a high number of presential visits. Future effectiveness studies could consider a follow-up medical visit at 4 months as it may increase the retention of patients.

This study is part of a continuous development cycle, that will continue with its multiple improvements and evaluation phases grounded on the interaction with end-users, aiming to produce a patient-centered and engaging mHealth asthma app.

In conclusion, the InspirerMundi app was feasible to monitor inhaler adherence in real-world patients with asthma. The persistent use of this mHealth technology varies widely. A better understanding of characteristics related to higher app use is still needed before effectiveness studies are undertaken.

## Data Availability Statement

The raw data supporting the conclusions of this article will be made available by the authors, without undue reservation.

## Ethics Statement

The studies involving human participants were reviewed and approved by Centro Hospitalar e Universitário São João and by the other participating centers. Written informed consent to participate in this study was provided by the participants' legal guardian/next of kin when patients were adolescents.

## Author Contributions

All authors listed have made a substantial, direct and intellectual contribution to the work, and approved it for publication.

## Conflict of Interest

The authors declare that the research was conducted in the absence of any commercial or financial relationships that could be construed as a potential conflict of interest.
